# Biomarkers of Kidney Tubule Disease and Risk of End-Stage Kidney Disease in Persons With Diabetes and CKD

**DOI:** 10.1016/j.ekir.2022.03.033

**Published:** 2022-04-05

**Authors:** Jonathan G. Amatruda, Ronit Katz, Mark J. Sarnak, Orlando M. Gutierrez, Jason H. Greenberg, Mary Cushman, Sushrut Waikar, Chirag R. Parikh, Jeffrey R. Schelling, Manasi P. Jogalekar, Joseph V. Bonventre, Ramachandran S. Vasan, Paul L. Kimmel, Michael G. Shlipak, Joachim H. Ix, CKD Biomarkers Consortium

**Affiliations:** 1 Division of Nephrology, Department of Medicine, University of California San Francisco, San Francisco, California, USA; 2 Kidney Health Research Collaborative, San Francisco VA Medical Center and University of California, San Francisco, San Francisco, California, USA; 3 Department of Obstetrics and Gynecology, University of Washington, Seattle, Washington, USA; 4 Division of Nephrology, Department of Medicine, Tufts Medical Center, Boston, Massachusetts, USA; 5 Department of Medicine, University of Alabama at Birmingham, Birmingham, Alabama, USA; 6 Department of Epidemiology, University of Alabama at Birmingham, Birmingham, Alabama, USA; 7 Section of Nephrology, Department of Pediatrics, Clinical and Translational Research Accelerator, Yale University School of Medicine, New Haven, Connecticut, USA; 8 Department of Medicine, Larner College of Medicine, University of Vermont, Burlington, Vermont, USA; 9 Section of Nephrology, Department of Medicine, Boston Medical Center, Boston, Massachusetts, USA; 10 Division of Nephrology, Department of Medicine, Johns Hopkins University, Baltimore, Maryland, USA; 11 Division of Nephrology, Department of Internal Medicine, MetroHealth System, Cleveland, Ohio, USA; 12 Department of Physiology and Biophysics, Case Western Reserve University School of Medicine, Cleveland, Ohio; 13 Division of Renal Medicine, Department of Medicine, Brigham and Women’s Hospital, Harvard Medical School, Boston, Massachusetts, USA; 14 Department of Medicine, Boston University School of Medicine, Boston, Massachusetts, USA; 15 Department of Epidemiology, Boston University School of Public Health, Boston, Massachusetts, USA; 16 National Institute of Diabetes and Digestive and Kidney Diseases, National Institutes of Health, Bethesda, Maryland, USA; 17 Department of Medicine, San Francisco VA Health Care System, San Francisco, California, USA; 18 Division of Nephrology and Hypertension, Department of Medicine, University of California San Diego, San Diego, California, USA; 19 Nephrology Section, Veterans Affairs San Diego Healthcare System, San Diego, California, USA

**Keywords:** biomarkers, chronic kidney disease, diabetes mellitus, end-stage kidney disease

## Abstract

**Introduction:**

Tubulointerstitial damage in diabetes and chronic kidney disease (CKD) is poorly captured by estimated glomerular filtration rate (eGFR) and albuminuria. Urine biomarkers of kidney health may better elucidate disease progression in persons with diabetes and CKD.

**Methods:**

Per case-cohort design, we randomly selected a subcohort of 560 study participants of the REasons for Geographic And Racial Differences in Stroke (REGARDS) study from 1092 adults with diabetes and baseline eGFR <60 ml/min per 1.73 m^2^ and registered a total of 161 end-stage kidney disease (ESKD) cases (*n* = 93 from the subcohort; *n* = 68 from outside the subcohort) during 4.3 ± 2.7 years mean follow-up. We measured urine biomarkers of kidney tubule injury (kidney injury molecule—1 [KIM-1]), inflammation and fibrosis (monocyte chemoattractant protein—1 [MCP-1]), repair (chitinase-3–like protein 1 [YKL-40]), and tubule function, including reabsorption (alpha-1-microglobulin [α1m]) and synthetic capacity (epidermal growth factor [EGF] and uromodulin [UMOD]). Weighted Cox regression models estimated ESKD risk adjusting for demographics, ESKD risk factors, and baseline eGFR and urine albumin. Least absolute shrinkage and selection operator (LASSO) regression identified a subset of biomarkers most strongly associated with ESKD.

**Results:**

At baseline, subcohort participants had mean age of 70 ± 9 years, mean eGFR of 40 ±13 ml/min per 1.73 m^2^, and median urine albumin-to-creatinine ratio of 33 (interquartile range 10–213) mg/g. Adjusting for baseline eGFR and albuminuria, each 2-fold higher urine KIM-1 (hazard ratio = 1.43 [95% CI: 1.17–1.75]), α1m (hazard ratio = 1.47 [1.19–1.82]), and MCP-1 (hazard ratio = 1.27 [1.06–1.53]) were independently associated with ESKD. LASSO retained KIM-1 and α1m for associations with ESKD.

**Conclusion:**

Among adults with diabetes and eGFR <60 ml/min per 1.73 m^2^, higher urine KIM-1, α1m, and MCP-1 are independently associated with incident ESKD, providing insight into kidney disease progression in persons with diabetes and CKD.

## Graphical abstract

**Figure:**
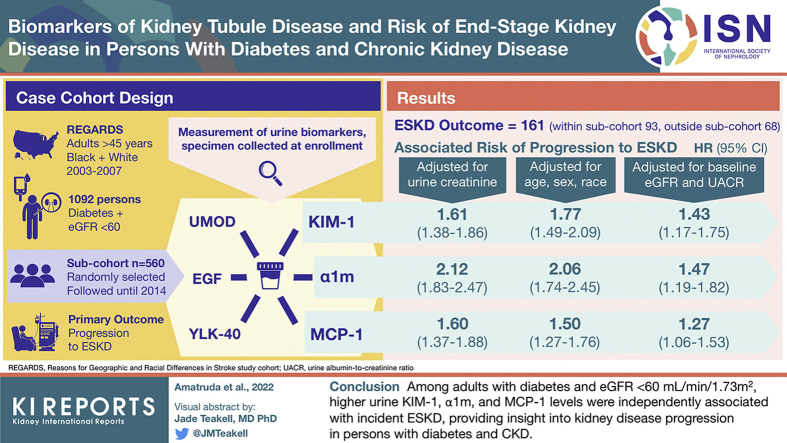

See Commentary on Page 1458

Diabetes is the leading cause of ESKD in the United States and a major contributor to the global burden of CKD.^1^^,^^2^ However, the risk of progressing from CKD to ESKD is heterogeneous among individuals with diabetes, and biological pathways leading to CKD progression remain uncertain.^3^

Although substantial research has focused on the glomerular sequelae of diabetes, pathologic alterations in the kidney tubules and interstitium have also been widely recognized.^4^^,^^5^ Importantly, histologic features of interstitial fibrosis, tubular atrophy, and inflammation on kidney biopsy are strongly associated with progression to ESKD in diabetes and may predict progression better than glomerular histology.^6^^,^^7^ Unfortunately, kidney biopsy is invasive and carries important risks, but clinicians and researchers currently lack alternative tests specific to tubulointerstitial health. Clinical diagnosis and risk stratification of kidney disease rely on eGFR and urine albumin-to-creatinine ratio (UACR), both of which primarily reflect glomerular function and integrity.^8^ These measures fail to fully capture kidney tubule health and, compared with biomarkers specific to tubule injury and inflammation, appear less sensitive for early and evolving tubulointerstitial disease.^9^^,^^10^ Investigational biomarkers of tubulointerstitial injury, fibrosis, and tubule function are currently under development, but their roles as indicators of progression to ESKD in diabetes are unclear. As compared with blood biomarkers, urine-based biomarkers of kidney tubule health are of particular interest as they may more directly reflect tubule health without confounding by systemic processes.^11^ In addition, urine is readily collected in both clinical and research settings, conferring substantial practical advantages.

In this study, we investigated 6 urine biomarkers specific to kidney tubule health, which are as follows: KIM-1, MCP-1, YKL-40, EGF, α1m, and UMOD. Together, these biomarkers capture multiple dimensions of kidney tubule health, including tubule injury (KIM-1), tubulointerstitial inflammation and fibrosis (MCP-1), tubule epithelial cell repair (YKL-40), tubule function including proximal tubule reabsorptive capacity (α1m), and tubule synthetic function (UMOD and EGF). Our goal was to determine whether these urine biomarkers inform risk of progression to ESKD independently of eGFR, UACR, and clinical risk factors among participants with diabetes and eGFR <60 ml/min per 1.73 m^2^.

## Methods

### Population and Study Design

The REGARDS study enrolled Black and White adults aged ≥45 years between January 2003 and October 2007 from across the continental United States.^12^^,^^13^ In total, 30,239 participants were recruited; all participated in a telephone interview followed by an in-home visit where they provided demographics and medical history, a physical examination, and blood and spot urine specimens.^12^^,^^13^ All participants provided informed consent, and the study was approved by the institutional review boards of all participating institutions. This ancillary study was in addition approved by the institutional review boards of Veterans Affairs San Diego and University of California, San Francisco.

We used a case-cohort design to study the relationship of biomarkers with risk of ESKD.^14^ First, we restricted the parent REGARDS study sample to the 1092 participants with diabetes and eGFR <60 ml/min per 1.73 m^2^ and without prevalent ESKD at the baseline visit. Among these 1092 participants, a case-cohort sample was selected with follow-up through June 2014 by randomly selecting a subcohort of 600 participants with baseline diabetes and eGFR <60 ml/min per 1.73 m^2^ and capturing all ESKD cases by linkage to the United States Renal Data System.^15^ In total, 174 participants meeting baseline criteria of diabetes and eGFR <60 ml/min per 1.73 m^2^ developed incident ESKD, 99 of whom were members of the random subcohort.

### Biomarkers of Kidney Tubule Health

Urine biomarkers for this study were selected by an expert panel of CKD Biomarkers Consortium members in the preinvestigation stage based on prior work. Urine was collected at baseline and centrifuged, and the supernatant was aliquoted with unique barcodes.^16^ Aliquots were kept in continuous laboratory storage at −80 °C until biomarker measurements were made. Personnel conducting biomarker measurements were blinded to clinical outcomes. Urine KIM-1, MCP-1, YKL-40, and EGF were measured on the Luminex platform with a laboratory-developed multiplex assay (Luminex Corporation, Austin, TX). UMOD was measured on the MSD R-PLEX (Meso Scale Diagnostics, LLC, Rockville, MD). Urine α1m was measured on a Siemens BNII nephelometer (Siemens, Inc., Munich, Germany). All measurements except α1m were made in duplicate, and mean values were used in the analyses. If intra-assay coefficient of variation exceeded 15%, the assay was repeated. All UMOD assays were performed with a single lot, which is notable because lot-to-lot variation of UMOD measurements on the MSD R-PLEX can be up to 50%.

### Covariates

Serum creatinine concentration was used to calculate eGFR according to the CKD Epidemiology Collaboration equation.^17^ Serum creatinine concentration was calibrated to isotope dilution using mass spectrometry. Urine albumin concentration was measured with the BNII ProSpec (Siemens, Inc., Munich, Germany). Urine creatinine concentration was measured by the Jaffe method on the Modular P chemistry analyzer (Roche/Hitachi, Basel, Switzerland). We adjusted for urine albumin and urine creatinine concentrations separately in multivariable models, whereas albuminuria was expressed as UACR in descriptive statistics.^18^

Sociodemographics and aspects of medical history were self-reported at the baseline interview. Prevalent cardiovascular disease was defined as self-reported stroke, myocardial infarction, coronary artery bypass graft, angioplasty, arterial stenting, or as evidence of past myocardial infarction on electrocardiography. Blood pressure was defined as the average of 2 measures taken on seated participants after a 5-minute rest. Use of medications for hypertension was obtained by self-report. Body mass index was determined using measured height and weight.

### Statistical Analysis

We tabulated descriptive statistics using baseline data and then calculated correlations between the urine biomarkers (KIM-1, MCP-1, YKL-40, EGF, α1m, UMOD), urine albumin, urine creatinine, and eGFR. Risk of ESKD was modeled using a time-to-event analysis with multivariable Cox regressions modified to account for the case-cohort design.^19^^,^^20^ We used Prentice’s original pseudolikelihood method, weighted such that risk sets at event times consist of subcohort members at risk whereas the cases outside the subcohort enter the risk sets only at their event times.^14^

Biomarkers were modeled continuously after log_2_ transformation and as quartiles. The main models focused on the log_2_-transformed biomarkers, whereas biomarker quartiles were used primarily to evaluate the functional form of associations. Values of α1m that were below the lower limit of detection were set to 5.47 mg/l. No other biomarker had values below the lower limit of detection. Quartiles of each biomarker were defined based on baseline concentrations in the subcohort sample. There were 3 staged models applied: model 1 adjusted only for urine creatinine concentration to account for differences in urine tonicity at the time of urine collection; model 2 additionally adjusted for age, sex, race, systolic blood pressure, antihypertensive medication use, body mass index, and prevalent cardiovascular disease; model 3 additionally adjusted for baseline eGFR and urine albumin.

Next, we performed LASSO regression to identify the biomarkers that retained independent associations with progression to ESKD when all biomarkers were simultaneously included in the model. This method penalizes the absolute size of regression coefficients and allows some parameter estimates to shrink to zero to produce a smaller set of the most predictive biomarkers while mitigating the risk of overfitting. To estimate penalized parameters, we used LASSO penalty with leave-one-out cross-validation.

All analyses were conducted using SPSS version 26.0 (IBM Corp., Armonk, NY) and R version 4.0.2 (R Foundation for Statistical Computing, Vienna, Austria). *P* < 0.05 was considered statistically significant.

## Results

**Figure 1:**
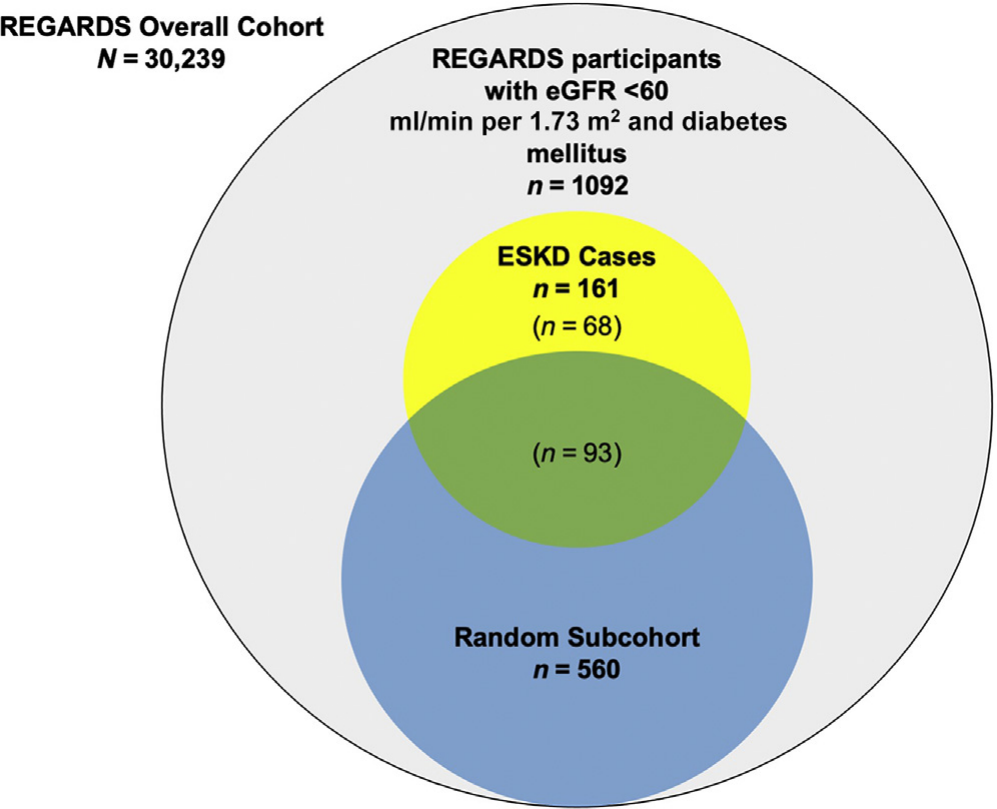
Sampling of REGARDS cohort per case-cohort design. Among 30,239 REGARDS participants, a total of 1092 had eGFR <60 ml/min per 1.73 m^2^ and diabetes at baseline, and a subcohort of 560 individuals with available baseline urine samples was randomly selected from those participants. There were 161 cases of incident ESKD, 93 of whom had also been selected into the subcohort and 68 cases arising outside the subcohort. eGFR, estimated glomerular filtration rate; ESKD, end-stage kidney disease; REGARDS, REasons for Geographic And Racial Differences in Stroke.

After excluding individuals with inadequate urine samples, the random subcohort was reduced to 560 persons (93%) and the total number of incident ESKD cases was reduced to 161 (93%), with 93 cases arising from the subcohort and 68 additional cases arising from outside the subcohort (Figure 1). Baseline characteristics of the subcohort and additional cases are presented in Table 1. In comparison to subcohort participants overall, ESKD cases arising from outside the subcohort were younger, more often self-identified as Black race, and had higher prevalence of hypertension, lower mean eGFR, and higher median UACR. These individuals also had higher average concentrations of kidney tubule health biomarkers except for EGF, for which average baseline concentrations were lower.

**Table 1:** Baseline characteristics of the REGARDS subcohort and additional ESKD cases

Characteristics	Subcohort (*n* = 560)	Additional cases (*n* = 68)
Age, yr	70 (9)	66 (8)
Men	263 (47%)	29 (43%)
Black race	299 (53%)	50 (70%)
Education		
Less than high school	123 (22%)	15 (22%)
High school graduate	152 (27%)	17 (25%)
Some college	143 (26%)	18 (27%)
College graduate and above	142 (25%)	18 (27%)
Insured	542 (97%)	61 (90%)
Body mass index	31.9 (6.6)	32.9 (7.6)
Hypertension	491 (88%)	65 (96%)
SBP, mm Hg	133 (19)	135 (17)
DBP, mm Hg	71 (11)	75 (11)
Heart failure	232 (41%)	35 (52%)
Coronary artery disease	225 (40%)	28 (41%)
Stroke	88 (16%)	12 (18%)
Antihypertensive use	469 (84%)	64 (94%)
ACEi/ARB use	415 (74%)	50 (74%)
Diuretic use	377 (67%)	45 (66%)
eGFR, ml/min per 1.73 m^2^	40 (13)	29 (11)
UACR, mg/g	33 [10–213]	424 [59–1607]
<30	270 (48%)	13 (18%)
30–300	164 (29%)	17 (25%)
≥300	126 (23%)	38 (56%)
Urine biomarkers		
KIM-1, pg/ml	1769 [1014–3476]	2103 [1138–4033]
EGF, pg/ml	1018 [768–1359]	727 [528–953]
YKL-40, pg/ml	330 [152–1074]	525 [129–2865]
MCP-1, pg/ml	215 [130–386]	285 [145–470]
α1m, mg/l	17 [8–32]	26 [15–55]
UMOD, μg/ml	3.9 [2.0–6.9]	6.2 [3.1–11.3]

α1m, alpha-1-microglobulin; ACEi, angiotensin-converting enzyme inhibitor; ARB, angiotensin receptor blocker; DBP, diastolic blood pressure; EGF, epidermal growth factor; eGFR, estimated glomerular filtration rate; ESKD, end-stage kidney disease; KIM-1, kidney injury molecule-1; MCP-1, monocyte chemoattractant protein-1; Q, quartile; REGARDS, REasons for Geographic And Racial Differences in Stroke; SBP, systolic blood pressure; UACR, urine albumin-to-creatinine ratio; UMOD, uromodulin; YKL-40, chitinase-3-like protein 1.

Data presented as mean (SD), number (%), or median [Q1–Q3].

Correlations of the urine biomarkers with each other and with urine albumin, urine creatinine, UACR, and eGFR in the random subcohort are shown in Table 2. Urine biomarkers were at most moderately correlated with each other and with eGFR and UACR, with correlation coefficients ranging from 0.1 to 0.5. In general, the urine tubule health biomarkers were positively correlated with each other and with UACR but negatively correlated with eGFR. However, EGF and UMOD demonstrated the opposite pattern of correlations. Urine EGF also had the strongest correlations with UACR and eGFR among the investigated biomarkers (−0.49 and 0.59, respectively).

**Table 2:** Correlations between investigational urine biomarkers and traditional measures of kidney health in the subcohort

	KIM-1	EGF	YKL-40	MCP-1	UMOD	α1m	Urine albumin	Urine creatinine	UACR	eGFR
KIM-1	1	0.151^a^	0.302^a^	0.649^a^	0.010	0.512^a^	0.441^a^	0.566^a^	0.280^a^	−0.032
EGF		1	−0.149^a^	0.078	0.394^a^	−0.243^a^	−0.314^a^	−0.506^a^	−0.448^a^	0.587^a^
YKL-40			1	0.336^a^	−0.370^a^	0.453^a^	0.439^a^	0.038	0.423^a^	−0.287^a^
MCP-1				1	−0.026	0.447^a^	0.389^a^	0.492^a^	0.249^a^	−0.090^b^
UMOD					1	−0.275^a^	−0.303^a^	0.345^a^	−0.393^a^	0.397^a^
α1m						1	0.664^a^	0.246^a^	0.588^a^	−0.420^a^
Urine albumin							1	0.090^b^	0.962^a^	−0.369^a^
Urine creatinine								1	−0.185^a^	0.184^a^
UACR									1	−0.413^a^
eGFR										1

α1m, alpha-1-microglobulin; EGF, epidermal growth factor; eGFR, estimated glomerular filtration rate; KIM-1, kidney injury molecule-1; MCP-1, monocyte chemoattractant protein-1; UACR, urine albumin-to-creatinine ratio; UMOD, uromodulin; YKL-40, chitinase-3-like protein 1.

a Correlation is significant at the 0.01 level (2-tailed).

b Correlation is significant at the 0.05 level (2-tailed).

### Association of Urine Biomarkers With Incident ESKD

Adjusting only for urine creatinine concentration, there were statistically significant associations between all the kidney tubule health biomarkers with the risk of ESKD (Table 3) when modeled as continuous measures. Higher urine concentrations of each biomarker were associated with risk of ESKD except for EGF and UMOD, in which the direction was opposite. Risk of ESKD rose 11-fold comparing the highest to the lowest quartile of α1m in the analysis adjusted for urine creatinine concentration alone. In contrast, risk of ESKD was approximately 10-fold lower for the highest quartiles of EGF and UMOD compared with the lowest. KIM-1, YKL-40, and MCP-1 demonstrated 4- to 5-fold increments in risk across quartiles in this model.

**Table 3:** Association of urine biomarkers with incident ESKD in REGARDS participants with eGFR <60 ml/min per 1.73 m^2^ and diabetes at baseline

KIM-1	Per 2-fold higher	Quartile 1: <1016 pg/ml	Quartile 2: 1016–1775 pg/ml	Quartile 3: 1776–3464 pg/ml	Quartile 4: >3465 pg/ml
Events/*N*	161/628	30/154	38/154	41/160	52/160
Model 1^a^	1.61 (1.38–1.86)	1.00 (ref)	1.99 (1.17–3.39)	2.31 (1.35–3.95)	4.70 (2.67–8.25)
Model 2^b^	1.77 (1.49–2.09)	1.00 (ref)	1.82 (1.04–3.19)	2.14 (1.20–3.83)	5.80 (3.16–10.66)
Model 3^c^	1.43 (1.17–1.75)	1.00 (ref)	1.41 (0.78–2.53)	1.35 (0.72–2.52)	3.06 (1.55–6.04)

MCP-1	Per 2-fold higher	Quartile 1: <130 pg/ml	Quartile 2: 130–216 pg/ml	Quartile 3: 217–385 pg/ml	Quartile 4: >385 pg/ml
Events/*N*	161/628	30/152	40/159	34/156	57/161
Model 1^a^	1.60 (1.37–1.88)	1.00 (ref)	1.84 (1.08–3.12)	2.02 (1.15–3.52)	4.80 (2.77–8.29)
Model 2^b^	1.50 (1.27–1.76)	1.00 (ref)	1.70 (0.98–2.97)	1.90 (1.07–3.40)	4.18 (2.35–7.46)
Model 3^c^	1.27 (1.06–1.53)	1.00 (ref)	1.77 (0.99–3.16)	1.86 (1.01–3.40)	2.23 (1.13–4.38)

YKL-40	Per 2-fold higher	Quartile 1: <169 pg/ml	Quartile 2: 169–416 pg/ml	Quartile 3: 417–958 pg/ml	Quartile 4: >958 pg/ml
Events/*N*	161/628	33/158	19/149	37/157	72/164
Model 1^a^	1.33 (1.24–1.43)	1.00 (ref)	0.72 (0.39–1.31)	1.32 (0.79–2.20)	4.16 (2.59–6.69)
Model 2^b^	1.27 (1.18–1.38)	1.00 (ref)	0.73 (0.39–1.36)	1.17 (0.68–1.99)	3.45 (2.07–5.75)
Model 3^c^	1.08 (0.99–1.19)	1.00 (ref)	0.80 (0.42–1.53)	1.07 (0.61–1.91)	1.84 (1.02–3.32)

EGF	Per 2-fold higher	Quartile 1: <767 pg/ml	Quartile 2: 767–1017 pg/ml	Quartile 3: 1018–1358 pg/ml	Quartile 4: >1358 pg/ml
Events/*N*	161/628	89/178	38/156	22/149	12/145
Model 1^a^	0.36 (0.28–0.46)	1.00 (ref)	0.34 (0.22–0.52)	0.17 (0.10–0.29)	0.09 (0.05–0.18)
Model 2^b^	0.40 (0.30–0.52)	1.00 (ref)	0.39 (0.24–0.64)	0.16 (0.09–0.28)	0.12 (0.06–0.25)
Model 3^c^	0.80 (0.57–1.12)	1.00 (ref)	0.64 (0.38–1.08)	0.35 (0.18–0.66)	0.34 (0.15–0.76)

α1m	Per 2-fold higher	Quartile 1: <8.25 mg/l	Quartile 2: 8.25–16.60 mg/l	Quartile 3: 16.61–32.10 mg/l	Quartile 4: >32.10 mg/l
Events/*N*	161/628	15/148	26/155	40/158	80/167
Model 1^a^	2.12 (1.83–2.47)	1.00 (ref)	2.18 (1.11–4.29)	4.43 (2.32–8.44)	11.10 (6.03–20.44)
Model 2^b^	2.06 (1.74–2.45)	1.00 (ref)	2.10 (1.04–4.22)	4.09 (2.07–8.80)	9.51 (4.90–18.48)
Model 3^c^	1.47 (1.19–1.82)	1.00 (ref)	1.72 (0.83–3.58)	2.68 (1.34–5.37)	3.34 (1.59–7.03)

UMOD	Per 2-fold higher	Quartile 1: <3.1 μg/ml	Quartile 2: 3.1–6.2 μg/ml	Quartile 3: 6.21–11.3 μg/ml	Quartile 4: >11.3 μg/ml
Events/*N*	161/628	67/163	47/165	37/157	10/143
Model 1^a^	0.71 (0.65–0.78)	1.00 (ref)	0.59 (0.39–0.88)	0.43 (0.29–0.66)	0.13 (0.06–0.27)
Model 2^b^	0.74 (0.65–0.84)	1.00 (ref)	0.62 (0.25–1.54)	0.46 (0.18–1.15)	0.16 (0.02–1.32)
Model 3^c^	1.00 (0.83–1.20)	1.00 (ref)	1.17 (0.69–1.98)	1.27 (0.50–3.23)	0.52 (0.19–1.44)

Urine albumin	Per 2-fold higher	Quartile 1: <12 mg/l	Quartile 2: 12–33 mg/l	Quartile 3: 34–203.5 mg/l	Quartile 4: >203 mg/l
Events/*N*	161/628	13/154	12/141	29/152	107/181
Model 1^a^	1.49 (1.40–1.58)	1.00 (ref)	1.38 (0.62–3.07)	3.58 (1.83–7.00)	16.44 (9.13–29.61)
Model 2^b^	1.47 (1.30–1.67)	1.00 (ref)	1.60 (0.70–3.66)	3.80 (1.88–7.70)	14.84 (7.74–28.43)
Model 3^c^	1.34 (1.23–1.47)	1.00 (ref)	1.47 (0.62–3.47)	2.72 (1.29–5.76)	9.07 (4.40–18.67)

α1m, alpha-1-microglobulin; EGF, epidermal growth factor; eGFR, estimated glomerular filtration rate; ESKD, end-stage kidney disease; KIM-1, kidney injury molecule-1; MCP-1, monocyte chemoattractant protein-1; *N*, number at risk; ref, reference; REGARDS, REasons for Geographic And Racial Differences in Stroke; UMOD, uromodulin; YKL-40, chitinase-3-like protein 1.

a Adjusted for urine creatinine concentration.

b Additionally adjusted for age, sex, race systolic blood pressure, body mass index, antihypertensive medication use, cardiovascular disease.

c Additionally adjusted for baseline eGFR and urine albumin concentration.

Adjustment for demographics and traditional CKD risk factors only minimally influenced the associations, all of which remained statistically significant in continuous analyses (Table 3). Additional adjustment for eGFR and urine albumin concentration substantially attenuated the associations for all biomarkers studied; however, higher α1m, KIM-1, and MCP-1 each remained significantly associated with risk of ESKD in continuous models. In these maximally adjusted models, each 2-fold higher concentration of α1m was associated with a 47% higher risk of ESKD, KIM-1 with a 43% higher risk of ESKD, and MCP-1 with a 27% higher risk of ESKD. For comparison, each 2-fold higher concentration of urine albumin was associated with a 34% higher risk of ESKD in this model.

**Figure 2:**
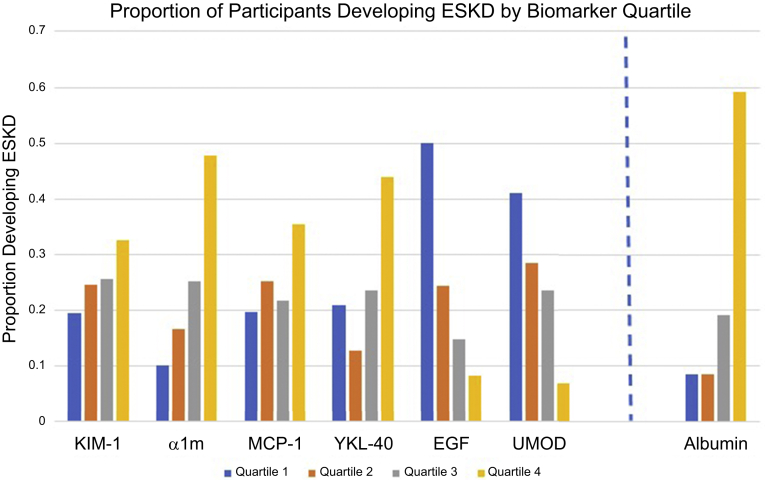
ESKD cases by quartile of each urine biomarker. α1m, alpha-1-microglobulin; EGF, epidermal growth factor; ESKD, end-stage kidney disease; KIM-1, kidney injury molecule-1; MCP-1, monocyte chemoattractant protein-1; UMOD, uromodulin; YKL-40, chitinase-3-like protein 1.

In analyses evaluating biomarkers by quartile, associations appeared monotonic except for YKL-40, in which the lower 3 quartiles had similar risk but the highest quartile had substantially higher risk of ESKD (Figure 2). Notably, the third and fourth quartiles of EGF were associated with a similarly reduced risk of ESKD compared with the first quartile (Table 3). The highest quartile of urine albumin concentration was associated with a 9-fold increment in ESKD risk in the fully adjusted model—a stronger association than any of those observed among the highest quartiles of the investigational biomarkers.

Finally, the LASSO regression model including all 6 tubule health biomarkers retained α1m and KIM-1, demonstrating associations of 36% and 31% higher risk per 2-fold higher urine α1m and KIM-1 concentration, respectively (Table 4).

**Table 4:** Associations of urine biomarkers with incident ESKD determined by LASSO regression

Per 2-fold higher level	Model 1^a^	Model 2^b^	Model 3^c^
	HR (95% CI)	HR (95% CI)	HR (95% CI)
KIM-1	1.14 (0.95–1.36)	1.28 (1.05–1.56)	1.31 (1.06–1.62)
α1m	2.02 (1.71–2.39)	1.84 (1.51–2.24)	1.36 (1.08–1.70)

α1m, alpha-1-microglobulin; CI, confidence interval; ESKD, end-stage kidney disease; HR, hazard ratio; KIM-1, kidney injury molecule-1; LASSO, Least Absolute Shrinkage and Selection Operator.

a Adjusted for urine creatinine concentration.

b Additionally adjusted for age, sex, race, systolic blood pressure, body mass index, antihypertensive medication use, and cardiovascular disease.

c Additionally adjusted for baseline estimated glomerular filtration rate and urine albumin concentration.

## Discussion

Among community-living adults with diabetes and eGFR <60 ml/min per 1.72 m^2^, urine biomarkers of tubule health were associated with risk of incident ESKD independent of clinical risk factors, eGFR, albuminuria, and the other tubule biomarkers. Although higher levels of urine α1m, KIM-1, and MCP-1 maintained associations with ESKD independent of other risk factors, LASSO regression identified α1m and KIM-1 as the 2 biomarkers most strongly associated with ESKD risk, independent of the other risk factors and one another. These findings indicate that one biomarker reflecting tubule reabsorptive dysfunction (α1m) and another reflecting tubule injury (KIM-1) provide complementary information on risk of ESKD along with glomerular function and injury in persons with diabetes and CKD. Notably, the associations of urine α1m and KIM-1 with incident ESKD were comparable to and independent of that of urine albumin concentration in adjusted continuous analyses. As albuminuria primarily indicates glomerular dysfunction, rather than tubulointerstitial disease, the combination of urine albumin with biomarkers specific to the kidney tubules could improve assessment of overall kidney health and potentially further add to the prognostic value offered by albuminuria. Moreover, these findings provide insight into the pathobiology of CKD in diabetes, supporting the importance of tubulointerstitial injury and dysfunction in kidney disease progression.

α1m is produced by hepatocytes, secreted into circulation, freely filtered across the glomerulus, and nearly completely reabsorbed by healthy proximal tubules.^21^ Exposure to drugs that cause proximal tubule dysfunction is associated with markedly higher urine α1m concentrations.^22^ Thus, higher urine α1m concentrations signify reduced proximal tubular reabsorptive capacity.^21^ We previously demonstrated that higher urine α1m concentrations are associated with kidney function decline in persons with HIV and in kidney transplant recipients.^23^^,^^24^ In the present study, we demonstrate similar findings among persons with prevalent CKD and diabetes.

Elevated urine KIM-1 concentration indicates proximal tubule cell injury.^25^ Because proximal tubule injury could be expected to cause dysfunction with decreased reabsorptive capacity, elevations in urine concentrations of both α1m and KIM-1 are consistent with a general phenotype of proximal tubule damage. However, only urine KIM-1 is presently qualified by the United States Food and Drug Administration as an early indicator of nephrotoxicity for use in preclinical drug development in animal models and humans.^26^^,^^27^ In a prior study of REGARDS participants with eGFR <60 ml/min per 1.73 m^2^ and UACR ≥30 mg/g, irrespective of diabetes status, higher urine KIM-1 level was independently associated with subsequent ESKD and death.^28^ The present analysis adds to this finding, demonstrating that KIM-1 provides additional information on risk of ESKD independent of α1m and the other 4 biomarkers. Furthermore, recent evidence suggests that in the diabetic kidney, KIM-1 may not only serve as a marker of injury but also contribute to its pathology by promoting proximal tubule cell damage via facilitating fatty acid uptake.^29^ Intracellular accumulation of these fatty acids results in oxidative stress and mitochondrial damage, initiating a cascade of proinflammatory and profibrotic responses.^29^ Sodium-glucose contransporter-2 inhibitors, which are recommended as first-line agents in diabetes and CKD to reduce risk of eGFR decline and ESKD, have been hypothesized to protect kidney tubules by blocking deleterious metabolic pathways.^30^^,^^31^^,^^32^ Notably, sodium-glucose co-transporter 2 inhibitor use has been associated with reductions in urine KIM-1 concentration in patients with diabetes, suggesting improved tubule health.^33^^,^^34^ Overall, these data support the overarching hypotheses that tubulointerstitial disease can be measured noninvasively and that these measurements inform the risk of progression to ESKD in high-risk individuals with diabetes and CKD, independently of those used in current clinical practice. Moreover, these findings highlight that measures of tubule function and injury provide complementary and independent insight into ESKD risk.

Several prior studies have evaluated novel biomarker approaches to characterize pathophysiology of kidney disease in persons with diabetes and risk factors for progression to ESKD. Though albuminuria has classically been considered the sine qua non of diabetic kidney disease, there is increasing recognition that many persons with diabetes and CKD do not have persistent or extreme elevations in urine albumin, even in cases of biopsy-diagnosed diabetic kidney disease.^35^, ^36^, ^37^, ^38^ These findings underscore the importance of identifying additional biomarkers, especially those that can reveal the accrual of tubulointerstitial damage. Using blood rather than urine, prior studies demonstrated that higher plasma concentrations reflecting both inflammation and tubule injury—particularly tumor necrosis factor receptor-1, tumor necrosis factor receptor-2, and plasma KIM-1—were independently associated with progression of kidney disease in diabetes.^39^^,^^40^ Our CKD Biomarkers Consortium collaborators have built on these prior studies, finding that plasma KIM-1, YKL-40, tumor necrosis factor receptor-1, and tumor necrosis factor receptor-2 were independently associated with incident ESKD among REGARDS participants with diabetes and CKD.^41^ The present study complements this work by reinforcing the importance of tubulointerstitial disease in persons with diabetes and CKD, but it has the important distinction of measuring novel biomarkers in urine rather than plasma. Plasma biomarkers necessitate venipuncture, may be influenced by systemic processes, and many are highly correlated with GFR. In contrast, urine is easily and painlessly collected, bolstering the utility of urine assays for both clinical and research applications. Furthermore, given their proximity to the kidney tubulointerstitium, urine biomarker concentrations are likely to correlate closely with disease activity and progression with less bias owing to GFR.^11^^,^^42^

These findings could potentially empower better monitoring of investigational agents in diabetes. An important question for future research will be whether higher urine α1M and KIM-1 concentrations could identify persons who would derive the most benefit from specific therapies aimed at the tubulointerstitium. Furthermore, these studies should investigate whether acute changes in these biomarkers after drug initiation inform the likelihood and magnitude of long-term treatment response. Other antifibrotic and antiinflammatory drugs are being investigated in CKD, and these biomarkers may have similar utility for patient selection and clinical monitoring.^43^, ^44^, ^45^

Our study has several strengths. The REGARDS cohort is large and provided a robust subcohort of persons with diabetes and CKD yielding >160 cases of incident ESKD during follow-up. The detailed measurements of demographics and comorbidities allowed extensive statistical adjustment for known ESKD risk factors. The highly efficient case-cohort design facilitated simultaneous evaluation of multiple urine biomarkers in an economical manner without sacrificing the ability to perform valid time-to-event analyses. This study also has important limitations, notably, that the observational design remains susceptible to confounding despite multivariable adjustment. The assays used to measure these biomarkers have not yet been standardized, which precludes the comparison of biomarker concentrations across studies for normative interpretation in clinical settings. Urine biomarkers were measured at a single time point; whether longitudinal changes in biomarkers are informative above and beyond their baseline concentrations remains uncertain. With a single measurement, higher concentrations of these urine biomarkers could theoretically have represented transient acute kidney injury episodes that were unrelated to risk of kidney disease progression; however, given that participants were asymptomatic volunteers in the ambulatory setting, it is unlikely that we captured substantial rates of incidental acute kidney injury. Whether these biomarkers could be uniquely informative in persons without albuminuria is of great interest, but we were unable to evaluate this question owing to the low rate of ESKD events in study participants with UACR <30 mg/g. It is also unknown whether or not the concentrations of these biomarkers may change in response to therapies that target tubulointerstitial inflammation and fibrosis. Studies aimed at answering these questions should be prioritized given the advent of promising new medications with the potential to prevent ESKD in persons with diabetes.

In conclusion, among community-dwelling persons with diabetes and eGFR <60 ml/min per 1.73 m^2^, higher urine α1m and KIM-1 concentrations were each strongly associated with incident ESKD, independent of clinical risk factors, eGFR, albuminuria, and one another. These findings support the hypothesis that tubule injury and dysfunction are important pathways of CKD progression in diabetes. That both α1m and KIM-1 provided information on risk of ESKD independent of one another demonstrates that quantification of both tubule injury and dysfunction may be complementary for discerning ESKD risk in individuals with diabetes and CKD, highlighting key pathways of kidney disease progression.^46^^,^^47^ Future research should establish whether these biomarkers provide opportunities for therapeutic monitoring of drugs targeting kidney inflammation and fibrosis.

## Disclosure

JHI has served on the data safety monitoring board for Sanifit and is the principal investigator of an investigator-initiated clinical trial supported by Baxter International. JHI reports consulting for AstraZeneca, Ardelyx, Akebia Therapeutics, and Jnana Therapeutics. CRP serves on the advisory boards of Renalytix AI, LLC, and GENFIT Pharmaceuticals. MGS has received funding from Bayer Healthcare Pharmaceuticals, Inc., and discloses consulting income from Intercept Pharmaceuticals, Inc., and Cricket Health. All the other authors declared no competing interests.
